# Unraveling the Underlying Heavy Metal Detoxification Mechanisms of *Bacillus* Species

**DOI:** 10.3390/microorganisms9081628

**Published:** 2021-07-30

**Authors:** Badriyah Shadid Alotaibi, Maryam Khan, Saba Shamim

**Affiliations:** 1 Department of Pharmaceutical Sciences, College of Pharmacy, Princess Nourah Bint Abdulrahman University, Riyadh 11671, Saudi Arabia; BSAlotaibi@pnu.edu.sa; 2 Institute of Molecular Biology and Biotechnology (IMBB), Defence Road Campus, The University of Lahore, Lahore 55150, Pakistan; maryamkkhan246@gmail.com

**Keywords:** bioremediation, *Bacillus*, resistance, microorganisms, biosorption, efflux, heavy metals

## Abstract

The rise of anthropogenic activities has resulted in the increasing release of various contaminants into the environment, jeopardizing fragile ecosystems in the process. Heavy metals are one of the major pollutants that contribute to the escalating problem of environmental pollution, being primarily introduced in sensitive ecological habitats through industrial effluents, wastewater, as well as sewage of various industries. Where heavy metals like zinc, copper, manganese, and nickel serve key roles in regulating different biological processes in living systems, many heavy metals can be toxic even at low concentrations, such as mercury, arsenic, cadmium, chromium, and lead, and can accumulate in intricate food chains resulting in health concerns. Over the years, many physical and chemical methods of heavy metal removal have essentially been investigated, but their disadvantages like the generation of chemical waste, complex downstream processing, and the uneconomical cost of both methods, have rendered them inefficient,. Since then, microbial bioremediation, particularly the use of bacteria, has gained attention due to the feasibility and efficiency of using them in removing heavy metals from contaminated environments. Bacteria have several methods of processing heavy metals through general resistance mechanisms, biosorption, adsorption, and efflux mechanisms. *Bacillus* spp. are model Gram-positive bacteria that have been studied extensively for their biosorption abilities and molecular mechanisms that enable their survival as well as their ability to remove and detoxify heavy metals. This review aims to highlight the molecular methods of *Bacillus* spp. in removing various heavy metals ions from contaminated environments.

## 1. Introduction to Heavy Metals

Living beings are constantly surrounded by different environments of land, air, and water, which are significant for their sustainability. The Earth is the fulcrum of all precious resources, where various fragile ecosystems work in harmony. Millions of years ago, the discovery of fire led to the revolutionary development of man, which culminated in the evolution and expansion of civilization. In the same manner, the dawn of industrialization saw the rapid growth and the impetuous exploitation of the Earth’s natural (renewable and non-renewable) sources, which gave rise to the predicament of environmental pollution [1]. This type of pollution has been associated with environmental contaminants of several kinds, such as organic and inorganic ions, isotopes, gases, nanoparticles, and organo-metallic compounds [2]. Regardless of these contaminants contributing greatly to environmental pollution, the presence of heavy metals in the environment is one point of grave concern over the past few decades.

According to Ali and Khan [3], heavy metals are defined as those naturally occurring metals “having an atomic number and density greater than 20 and 5 g/cm^−3^, respectively”. Even more so, the term “heavy metal” is now inclusive of the metallic elements as well as metalloids, both of which are somewhat toxic to the living beings and environment, including metalloids such as selenium (Se), arsenic (As), and tellurium (Te) [4]. Nevertheless, there are many metals which are not relatively toxic to both humans and the environment, including gold (Au) [5,6]. The toxicity of metals and metalloids is influenced by their ability to form covalent bonds, usually with organic groups resulting in the formation of lipophilic compounds distributed widely in the Earth’s biosphere, some of which are more toxic than the ionic form of the major metal found in those compounds, like tributyltin oxide in the case of arsenic. Heavy metals tend to facilitate their entry in human beings by one or more of the following methods: consumption of contaminated food and drinks; atmospheric inhalation; drinking of contaminated water; and/or direct contact with agricultural, pharmaceutical, residential, and industrial contamination [7]. They readily persist in the environment and are non-biodegradable. Many of the metals are detoxified by the action of living organisms concealing the element inside a protein or by mediating its deposition within intra-cellular granules which can then be stored or expelled accordingly. The gradual persistence of heavy metals in our ecosystem means that humans will eventually come into contact with them, which can potentially lead to their bioaccumulation in the human body. Bioaccumulation is the process through which contaminants, particularly heavy metals, are accumulated in the human body through ingestion or inhalation and can potentially be dangerous, as they tend to cause various physiological and biological complications. It is important to note here that this phenomenon is relative to the toxicity of heavy metals, which is usually observed in high concentrations [8]. The sources of heavy metals in the environment can comprise of geological, natural, lithogenic, and anthropogenic origins. Natural sources of heavy metals in the environment comprise of soil and rock erosion, volcanic eruptions, as well as sediment run-off [9]. Many anthropogenic processes also contribute to the presence of heavy metals in the environments, with rapid industrialization being the main culprit in this case. The release of heavy metals via insecticides, pesticides, and phosphate fertilizers lead to the release of metals ions such as zinc and cadmium. Moreover, metals such as mercury, arsenic, and lead have also been used in the agricultural fields, which leads to their accumulation in soil and underground sources, along with their accumulation in trophic levels of the food chain [10,11,12]. In addition, industrial processes such as metal and coal mining, fossil fuel combustion, and disposal of wastes and effluents has also greatly increased the burden of heavy metal pollution [13]. In water sources, rivers and streams that are near industries and mining areas tend to become polluted by heavy metals, speeding up their precipitation towards the sedimental level due to their decreasing solubility [14].

Contingent to their significance in biological systems, most of the heavy metals can be differentiated as essential and non-essential heavy metals, respectively. Essential heavy metals are characterized as necessary for biological life and its biochemical and physiological processes in low concentrations, as high concentrations can represent toxicity. On the other hand, non-essential heavy metals are those which have no reported biological function in the living systems, and only contribute to exert their toxicity in various biological beings. Examples of the former include heavy metals such as manganese, iron, copper, and zinc, while heavy metals like cadmium, lead, and mercury tend to be non-essential [15,16]. Moreover, heavy metals such as manganese, iron, cobalt, nickel, copper, zinc, and molybdenum are regarded as trace elements for the biochemical and biological growth of plants. These metals have also been recognized as essential for the development of growth and survival in stress environments, along with the biosynthesis of various organic compounds and metabolites [17]. Essential heavy metals are significant due to the fact that their deficit can lead to serious growth abnormality or stress for the living being. Nevertheless, the essential heavy metals required by every biological system (i.e., plants, animals, microorganisms, human beings) vary greatly from one to another, as the interaction of heavy metals with each of the organisms is reported to be an intricate process [18,19]. Organelles and cellular components such as mitochondria, cell membrane, and enzymes tend to be affected by heavy metal concentrations, as the interaction of these metal ions and cellular proteins has reported to cause DNA damage, as well as alteration of the normal cell cycle leading to apoptosis and often carcinogenesis [6]. The damage to DNA can be mediated by two mechanisms, namely direct and indirect damage, where the former refers to the conformational changes occurring in the biological molecules because of interaction with the metal ions, and the latter leads to the generation of reactive oxygen species, free radicals, and other oxidative stress species, respectively [20]. Heavy metals like iron, chromium, vanadium, cadmium, and copper are reported to be involved in the formation of free radicals by following the Fenton reaction of superoxide and hydroxyl ions, usually in the mitochondrial and peroxisomal region of cells [21].

## 2. Methods of Heavy Metal Remediation

When compared to organic pollutants, heavy metals are considered to be persistent in the environment because they are non-biodegradable and cannot be broken down easily. Incessant accumulation of heavy metals in soils may damage their ecosystem and inadvertently lead to the disturbance of various physicochemical properties of soils, such as pH, anion/cation exchange, thermal and electrical conductivity, microbial ecosystems, and the mobility of heavy metal ions in the soil [22]. Various studies have reported the accumulation of heavy metals in soils and their risks to biological systems. At high concentrations, heavy metals are able to directly and indirectly affect associated microbial ecosystems [23]. Thus, it is crucial to apply efficient technologies which are ultimately cheap, feasible, and are prone to site-specificity for the remediation of contaminated environments. Over the past two decades, many remediation techniques for soil and water have been developed and investigated which have proven to be efficacious in mitigating the total content of heavy metal ions and/or their accumulation at trophic levels in the food chain [23,24]. Physical methods such as heat treatment [25], soil replacement [26], soil washing [27], electroremediation [28] and vitrification [29,30] are employed for their widespread applications in the removal of various waste products. This aspect ensures the efficiency of physical methods in removing almost every kind of contaminant in the environment, though this property does not come without some disadvantages. The contaminants removed by physical methods need to be processed further, which adds to the already high cost of the method used, along with their specificity. Chemical methods for heavy metal remediation are also employed to alter the chemical characteristics of contaminants that subsequently decrease their hazardous properties. Though methods like precipitation [31], leaching [32], extraction [33], ion exchange [34], encapsulation [35], and immobilization [36] are observed to be efficient at field level, the generation of byproducts may act as a major setback which leads to additional processes at downstream level. Many of these techniques are contingent upon physical, chemical, and biological methods of bioremediation, which may be used singularly or in combination with each other for the same purpose. In spite of their effectiveness, many of these techniques are not feasible, environmentally-friendly, or cheap, which ultimately makes them challenging to be used. Nevertheless, new techniques are always being introduced in the search of the most suitable method of remediation.

## 3. Bioremediation—An Environmentally-Friendly Approach for the Removal of Heavy Metals

Bioremediation is a conventional process that employs the use of plants, animals, and microorganisms for the remediation of pollutants like heavy metals. It is one of most effective, non-invasive, and economically feasible methods for the permanent mitigation of heavy metal pollution and for the complete restoration of the natural environment of many ecosystems, as there is no production of secondary by-products [37]. Though methods such as phytoremediation (bioremediation using plants), phycoremediation (bioremediation using algae) and mycoremediation (bioremediation using fungi) have been widely reported in literature for heavy metal removal, our review focuses on bacterial bioremediation with *Bacillus* spp. as potentially active and efficacious agents for the removal of various heavy metal ions present in the environment.

Bacteria are abundantly present in the environment, where their variety in shape, size, morphology, as well as resistance mechanisms makes them suitable for bioremediation of organic and inorganic pollutants. Biosorption mediated by bacteria is a cost-effective, economically feasible method for the removal of heavy metal ions and other pollutants from contaminated sites, as many species have evolved to develop mechanisms which mediate resistance to process heavy metal ions amidst high concentrations [38]. Over the past years, many bacterial species have garnered widespread attention for their potential ability to remove heavy metal ions from the environment, whereas bacterial biomass (live and dead) has also been investigated for biosorption of metal ions such as copper, chromium, zinc, nickel, cadmium, and mercury, concomitant on their interactions with the bacterial cell wall and related peptidoglycans [39,40,41]. Many bacterial species such as *Bacillus*, *Pseudomonas*, *Enterobacter*, *Flavobacterium*, *Geobacter*, and *Micrococcus* spp. have been investigated for potential use in biosorption of various heavy metal ions from contaminated sites, by observing their surface to volume ratio and other feasible characteristics which enhance the binding interactions between bacterial functional groups and heavy metal ions [42,43]. In a recent study, the biosorption ability of many bacterial species such as *B. subtilis*, *B*. *megaterium*, *A. niger*, and *Penicillium* spp. were investigated for the removal of many heavy metal ions such as lead, chromium, and cadmium, of which *Bacillus* spp. were observed to be the most efficient [44,45].

## 4. *Bacillus* spp. as Potential Agents for Heavy Metal Removal and Their Overall Significance

*Bacillus* spp. are Gram-positive, rod-shaped bacteria belonging to the phylum Firmicutes, and are spore-forming in nature. They are generally characterized as soil microorganisms which can be aerobic or facultatively anaerobic but can also be found in various sources such as air, water, edible produce, foods, and the human gut [46]. In terms of features presented as genetic or commercial applications, *Bacillus* group is the most heterogenous as some species have been well characterized as opportunist pathogens and toxin producers, whereas others have widespread industrial and medicinal applications [47]. One of the unique characteristics of *Bacillus* spp. is the formation of spores under extreme environments, which is usually triggered during a deficit of nutrients [48,49]. Owing to their resilient structures, spores are able to endure severe environmental stress such as desiccation, high temperature, humidity, as well as radiation [50]. It is due to this feature they exhibit various commercial applications and are more suitable to be used than vegetative cells, respectively [51].

The positive use of *Bacillus* spp. in various fields has attracted attention to their characteristic features, and research has enabled the utilization of these features to the best of man’s interest. In aquaculture and fishery environments, *Bacillus* are utilized as biocontrol products [52]. In medical, industrial, and environmental fields, the advantage of using Gram-positive bacteria like *Bacillus* is that they do not tend to partake in the transfer of genetic material from Gram-negative bacteria. Moreover, they replicate expeditiously and can survive in many environmental conditions. Many species of *Bacillus,* such as *B. subtilis*, *B. coagulans*, *B. pumilus*, *B. licheniformis*, and *B. cereus* are utilized globally for many applications [51]. Many study findings have indicated the safety of *B. subtilis* for probiotic use, due to the demonstration of antimicrobial and anti-cancerous effects [53]. *Bacillus* spp. are also used for the production of various enzymes such as amylase, protease, cellulase, and pectinase in the food industry, [54] as well as in several supplemental nutrients such as vitamins and carotenoids [55,56]. Apart from these uses, *Bacillus* spp. are also extensively investigated for their role in the mitigation of heavy metals from contaminated environments via biosorption, bioaccumulation, among many other techniques, due to the reported notion that contaminated sites are often dominated by Gram-positive bacteria, owing to their versatile metabolic properties and better qualities of biosorption [57,58].

## 5. *Bacillus* Species and Heavy Metals

### 5.1. Arsenic

Arsenic (As) is a naturally occurring toxic metalloid [59] that is reported in soil (5–10 mg/kg), rock (1–2 mg/kg), sea water (1–3 µg/L) [60,61,62], as well as air and volcanic ash (0.02 µg/m^3^), due to which arsine gas (AsH_3_) and methylated arsine species enter into the surroundings [63]. Its anthropogenic sources are herbicides, pesticides [64], fossil fuel combustion, mining, smelting, wood preservation, sludge, manure [65], paint pigments, ceramic, glass industry, and food additives [66,67,68]. It exists as insoluble sulfides and sulfosalts [69]. It has four oxidation states: As^3-^ (arsine), As^0^ (elemental arsenic), As^3+^ (arsenite), and As^5+^ arsenate. Among them, As^3+^ and As^5+^ are the most abundant forms. As^3+^ is more toxic and mobile in aqueous and oxic environments than As^5+^, which is mostly adsorbed to the sediments in an anoxic state [70,71]. The histidine part of cellular proteins is a target site for the interaction of thiols of cysteine residues and/or imidazolium nitrogen with As^3+^, which results in the inactivation of enzymes [72]. As^5+^ interferes with protein synthesis by replacing the phosphate group during phosphorylation of the energy transfer process. The existence of either state depends on the redox state of the environmental conditions [73], as well as the geomicrobial population [74]. The US EPA and WHO has allowed 10 µg/L as the permissible concentration of As in drinking water [65]. Above 0.5 ppm, it is toxic for living systems resulting in symptoms of skin cancer [75], loss of appetite, weakness, weight loss, lethargy, chronic respiratory disorders, gastrointestinal disorders, enlargement of liver, spleen disorders, anemia [76], and cardiovascular disease [77]. Introduction of arsenic into the food chain and ground water may lead to serious concerns of arsenicosis [71].

#### Bioregulation of Arsenic by *Bacillus* spp.

**Figure 1:**
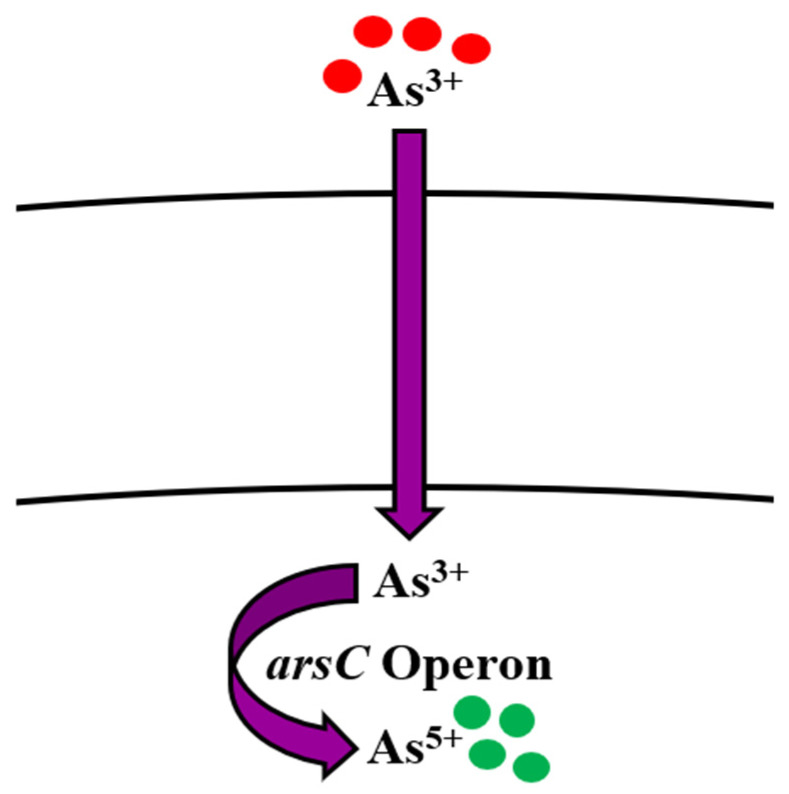
Bioregulation mechanism of arsenic in *Bacillus* spp.

The soluble nature of As makes its removal from the environment difficult [78]. Physical and chemical remediation of arsenic involves an oxidation step for converting As^3+^ into As^5+^. It may occur under atmospheric oxygen, which is usually very slow, or by using chemical oxidants like hydrogen peroxide, chlorine, or ozone. This method is very expensive and produces harmful byproducts [79]. Microorganisms use As as an energy source in their metabolic processes and thus transform the toxic As^3+^ into its less toxic form, As^5+^ [80,81] by arsenic oxidase, which is present in the protoplasm of arsenic-oxidizing bacteria [59]. Liao et al. [82] reported *Bacillus* as one the important arsenic-reducing bacteria. Arsenic removal by *Bacillus* spp. like *B. megaterium* [83,84], *B. aryabhattai* [85], and *B. cereus* strain W2 was studied by Miyatake and Hayashi [86]. Anaerobic respiration of As^5+^ by *B. cereus* was reported by Ghosh et al. [71]. Various other studies conducted over the years have reported the ability of *Bacillus* spp. to uptake As [87,88,89,90,91] (Table 1). As speciation, solubility, and mobilization are dependent on its methylation [92,93], oxidation [94], reduction, and respiration. The significance of oxidative phosphorylation in living organisms cannot be ignored [68]. Structurally, As^5+^ has similarity with phosphate [65]; thus, it is a potential oxidative phosphorylation inhibitor [68]. As^5+^ enters the organism’s living system by employing two pathways; Pit and Pst [94], which are usually used for phosphate uptake. It interferes with phosphorylation metabolic reactions and inhibits synthesis of adenosine triphosphate [59]. The portal of entry for As^3+^ is aquaglyceroporin proteins [65,95,96]. After internalization, it immediately binds to the respiratory enzymes via their sulfur residue [66,97]. Bioremediation of As in *Bacillus* spp. is done by *ars* operon [98] (Table 2) by employing three genes *arsA*, *arsB*, *arsC*, *arsD*, and *arsR*. *arsA* and *arsB* have ATPase activity, *arsC* transforms As^3+^ to As^5+^, *arsD* works as metallochaperone, while *arsR* acts as a repressor [99,100,101,102]. Normally, As^5+^ (less toxic form) that enters the cell is reduced to As^3+^ (more toxic form) by ArsC and then transported out of the cell by ArsB [103,104] (Figure 1).

### 5.2. Zinc

Zinc (Zn) is one of the most profusely abundant transition elements in the Earth’s crust, and is also widely found in biological systems, coming second only to iron. Zn (atomic number 30) is a member of group XII (previously known as II-B) of the periodic table of elements, and as analogous to all members of the group, it is also characterized as a divalent metal [199]. At room temperature, it occurs as a brittle, lustrous metal with a blue-white hue [200]. In reactions concerning hydrolysis, Zn tends to act as a Lewis acid or electrophile, which catalyzes these reactions and is thereby integrated into assorted metallo-enzymes, transcription factors, and regulatory proteins [201]. In cells, it exhibits antioxidative properties against the formation and mitigation of free radicals and reactive oxygen species, which contributes to the perpetuation of protein stability. The significance of Zn resonates with its function as an essential nutrient in living systems, augmenting its presence in both human beings and bacteria, where more than 5% of bacterial proteins evince their dependency on Zn [202]. These manifold functions preponderate over its toxicity at higher concentrations in cells, which can often be promoted by the blockage of protein thiols via mis-metallation with other metals, resulting in the disruption of various biological functions [203].

In the environment, anthropogenic actions have shaped the presence of Zn and its compounds in industrial and agricultural wastewaters, underscoring the production (more than 12 million tons annually) and then consumption of Zn in a multitude of processes such as galvanization, metallurgy, and the pharmaceutical industry, alloy metal casting, pesticides, and production of several other consumer goods [204]. Moreover, mining activities and contamination of sludge in soils poses a considerable threat of Zn toxicity to the sustainability and quality of crops [205], which further raises concern for the purification of contaminated sites by effective methods. It has been reported that the removal of Zn in low concentrations is mediated by physical and chemical methods, but its removal by biological agents (plants, algae, microorganisms) is a method which has been gaining attention due to its many advantages that eclipse its drawbacks. The treatment of Zn-contaminated wastewaters through plants, biomass, sawdust, mollusk shells, fruit and vegetable peels, agricultural wastes, and polysaccharides such as chitosan and pectin as potentially effective biosorbents has been widely reported [206,207].

#### Bioregulation of Zinc by *Bacillus* spp.

**Figure 2:**
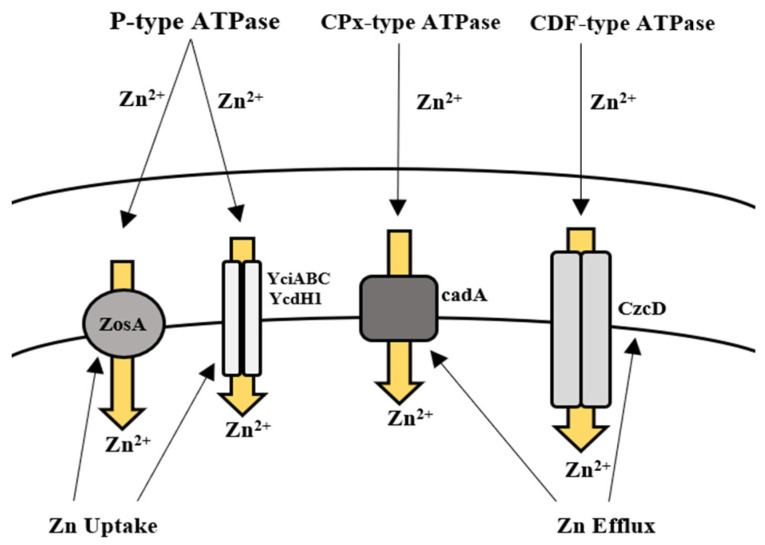
Uptake and efflux mechanism of *Bacillus* spp. for the regulation of zinc.

The action of *Bacillus* spp. in removing Zn from contaminated environments has been highlighted in many bioremediation studies. This ability of *Bacillus* spp. is regulated either by acquiring resistance through plasmids or by evolving mechanisms of resistance [208,209]. In *B. subtilis*, Zn uptake is regulated by the Zur family, which enables the transport of Zn ions via two transporter proteins. Gaballa et al. [157] also reported a third uptake system in *B. subtilis* for Zn ions called ZosA (P-type ATPase), which was expressed in conditions of oxidative stress. Efflux of Zn in high concentrations is facilitated by a CPx-type ATPase efflux pump in *B. subtilis*, known as CadA [157,158] (Table 2, Figure 2). Moreover, the removal of Zn by *Bacillus* sp. such as *B. subtilis*, *B. licheniformis*, *B. cereus*, *B. jeotgali*, and *B. firmus* was reported in recent studies [105,106,107,108,109,110,111] (Table 1). Khan et al. [112] also reported the removal of Zn (87 mg/L) by *B. altitudinis* isolated from industrial wastewater.

### 5.3. Nickel

Nickel (Ni) belongs to group 10 and is the 28th element in the periodic table, discovered by Swedish chemist Axel Cronstedt in its purified form for the first time in 1951. It is a hard, silvery-white transition metal which belongs to the ferromagnetic group of metals with high electrical and thermal conductivity [210]. It is the 24th most copious element found in the Earth’s crust, and the 5th most abundantly found in terms of weight. It is naturally found in its oxidation state (2+) which is analogous to most environmental and biological settings, though it may exhibit other valences as well (−1 to +4) [20]. It persists in nature in its hydroxide form at pH > 6.7, while its complexes appear to be readily soluble at pH < 6.5. It is found to exist in various forms of air- and water-resistant minerals (oxides and sulfides) [211], which give rise to Ni salts of strong (readily soluble in water) and weak acids (poorly soluble in water), respectively [212]. Natural sources of Ni in the environment are attributable to soil and rock erosion, volcanic eruptions, meteorite emissions, air-blown dust, as well as foods [213]. Combustion of fossil fuels and leaching from rocks and soil contribute to its presence in air and water, respectively. Moreover, anthropogenic emissions in the form of metal smelting and mining, metal refineries, Ni plating and alloy production, and effluent and sludge disposal into soil and water catalyze its presence in high concentrations in the environment [214]. The commercial use of Ni and its extensive applications such as production of Ni-Cd batteries, use in jewelry, orthodontic equipment, machinery, coins, food processing, clothing, and electronics promote its ubiquity in the environment, where it exists as sulfides, oxides, and less frequently, in its metallic form [215]. Ni toxicity has been the subject of widespread research in humans, where it is highlighted that the metal poses no considerable nutritional value in humans and poses industrial and occupational hazardous risk [216]. Nevertheless, it has been characterized as essential for the growth of plants, microorganisms and animals [217], where Ni-based enzymes and cofactors are reported to serve a key role in their function [218].

#### Bioregulation of Nickel by *Bacillus* spp.

**Figure 3:**
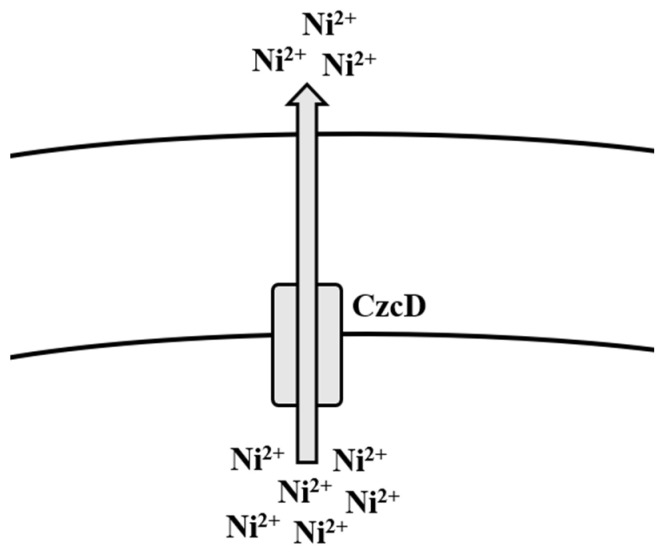
Efflux mechanism of nickel in *Bacillus* spp.

There have been many methods of Ni removal from solid matrices, but the most effective are those which are capable of removing/treating Ni before it emanates into the environment [219]. Several physico-chemical methods have been employed over the years for the removal of Ni from aqueous solutions [220]. Regardless of which of these methods have been used in the past, newer, cheaper, and more efficient methods of adsorption have been used for Ni removal, such as the use of biomass, where sugarcane, corn cobs, citrus peels, and bark have been used [221]. In a study, corn hydrochar was treated with KOH and altered by treating polyethyleneimine (PEI) to increase adsorption of Ni ions onto the surface [222]. Bioremediation by Gram-positive bacteria, such as *Bacillus* spp., has been the better method for the removal of Ni ions from Ni-contaminated media. There have been many studies that demonstrate the uptake and/or removal of Ni ions from contaminated environments such as soils, wastewater, and rivers [105,113,114,115] (Table 1). *B. thuringiensis* has also been frequently reported to uptake and remove Ni from contaminated environments [116,117,118]. In a recent study, *B. megaterium* was isolated from Ni-contaminated soils and was able to uptake more than 500 mg Ni, where more than 3000 mg/L Ni salt was previously found [223]. The removal of Ni by bacteria is contingent on their inherent mechanisms of resistance, which ultimately facilitate uptake, transportation, and efflux of the metal ions in and out of the cell. According to Moore et al. [159], mechanisms of Ni homeostasis and regulation have not been well characterized in *B. subtilis* when compared to Gram-negative bacteria such as *Escherichia coli* and *Helicobacter pylori*, though some evidence suggests their presence [224]. Members of the cation diffusion facilitator (CDF) family have long been characterized to mediate efflux of multiple metal ions, including Ni [160]. In *B. subtilis*, the cation diffusion transporter CzcD is reported to provide protection to the cell amid high concentrations of Ni^2+^, Cu^+^, Zn^2+^, and Co^2+^ [159] (Figure 3). When these ions happen to be bound with citrate, these complexes, during favorable conditions, are taken up by the metal-dicitrate uptake system known as CitM in *B. subtilis*, consequently leading to an increase in toxicity to them [159] (Table 2).

### 5.4. Cadmium

Cadmium (Cd) is a member of group XII of the periodic table and is a silvery white metal in appearance, with physical and chemical properties similar to both zinc and mercury. It exists in general oxidation state (+2) and is malleable and ductile. Cd is a corrosion-resistant metal, which is not flammable and water-soluble in nature, and is generally regarded as a toxic heavy metal with wide application in the industries of batteries, plating, plastics, and pigments, contributing greatly to its toxicity [218]. Anthropogenic activities have led to its presence being observed in many food sources and drinks [225]. Cadmium oxide is often used in metal plating, catalysis, and ceramic glaze. Alongside cadmium telluride, it has been also used in the form of a thin film for use in diodes, transistors, solar cells, electrodes, and anti-reflective coatings [226]. Cd is also used extensively in industrial strength paints, which can pose an environmental hazard during spraying. Other sources of contamination such as Cd-containing fertilizers can pollute soils which can inadvertently enhance its absorption by humans and other living beings. Cd has no known biological function and is a great threat to all life forms [227], due to which its environmental exposure can be dangerous, and in some cases, fatal [228].

#### Bioregulation of Cadmium by *Bacillus* spp.

**Figure 4:**
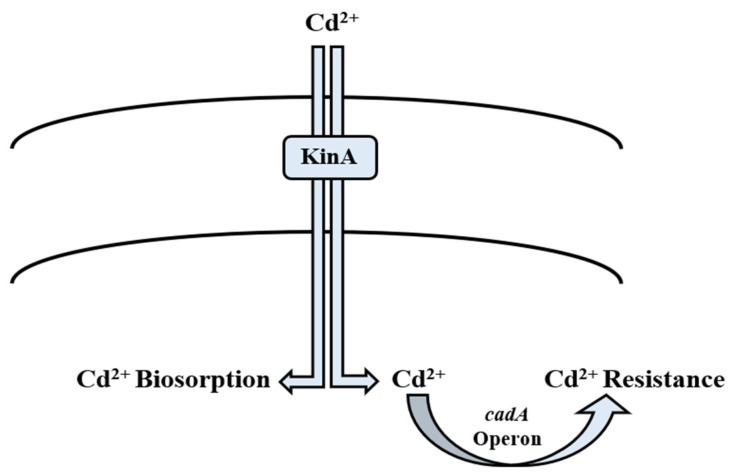
Mechanism of cadmium bioregulation by *Bacillus* spp.

Nature has gifted microorganisms with *cadA* operon to combat Cd toxicity. It is a 3.5 kb operon located on plasmid pI258. It has two genes; *cadA* and *cadC* [229,230]. *cadA* is transcribed into the 727-amino acid protein, which performs the function of energy-dependent Cd efflux ATPase [134], whereas *cadC* encodes relatively a small protein of 122 amino acids, and is a positive transcription regulator of Cd^2+^ operon [230,231]. It is well reported that *cadA* gene is induced in the presence of Cd^2+^ ions. Solovieva and Entian [161] documented their findings on *cadA* as a chromosomal determinant as well as a new gene *yvgW* of *B. subtilis* involved in Cd^2+^ resistance. Moreover, they reported that it has similarity to *cadA* of *S. aureus* plasmid pI258 [232]. Deletion of *yvgW* increased the Cd^2+^ sensitivity in the bacterium. *cadB* is also plasmid-mediated and confers Cd^2+^ resistance through a change in the binding site [162]. In addition to efflux mechanism, KinA and histidine kinase provide phosphate for phosphorylation which leads directly to transcription in *B. subtilis* [163]. Its overexpression results in phosphate flux of the cell, thereby directly affecting the energy state of the cell wall [233]. It also plays a significant role in biosorption of Cd^2+^ ions by phosphorylating the *Bacillus* cell surface magnitude, hence it acting as a major phosphate provider [122]. Cd^2+^ enters the bacterial cell membrane via the zinc and manganese transport systems, which are chromosomally mediated. Cd^2+^ concentration greatly affects the whole process. If the Cd^2+^ ions are present in high quantity in the medium, much extracellular adsorption is observed. Otherwise, intracellular Cd^2+^ concentration is high [234]. The *cad* operon of *Bacillus megaterium* TWSL_4 contains cadC, which has Cd^2+^ and Zn^2+^ metal binding motifs [235]. On exposure to metal ions, the first interaction is always with cell wall [236]. Its structure and composition play a significant role in deciding the next step of the process. Cd^2+^ adsorption on the bacterial cell wall deals with exchanging ions like Ca^2+^, Mg^2+^, and H^+^ ions [237]. Chelation is another process in which Cd^2+^ ions are exchanged with cell surface protons like –SO_3_H, –COOH, and –NH [238]. It involves sequestration via intracellular metallothionein (MT) [239]. Inorganic deposition of Cd^2+^ in the cell wall or inside cells can take place through interaction with hydroxide, carbonate, sulfate, and phosphorus [240]. In addition to metal concentration, biosorption is dependent on cell wall composition and cell physiology [241]. In Gram-positive bacterial species, resistance to Cd^2+^ is achieved by *cadA* system that is plasmid-borne. Cd^2+^ enters the bacterial cell by the MIT (metal ion transporter) system [164,165] (Table 2). The genes for Cd^2+^ resistance are mostly plasmid-mediated. According to Chen et al. [242] they are found on R plasmid along with antibiotic resistance genes, e.g., in pathogens including *K. pneumoniae*, *P. aeruginosa*, and *S. aureus*. These genes are reported to be directly involved in the uptake of Cd^2+^ ions from the environment (Figure 4). Basha and Rajaganesh [120] reported *B. licheniformis* to be a good biosorbent for Cd^2+^, as it removed more than 98% of Cd^2+^. Other species such as *B. catenulatus* and *B. safensis* are also reported to be effective in removing Cd^2+^ [119,121] (Table 1).

### 5.5. Lead

Lead (Pb) is a toxic heavy metal that is introduced into the environment via the weathering of rocks. Anthropogenic sources include fossil fuels, extraction and melting of metals, battery-manufacturing industries, insecticides, pigments, and fertilizers [243]. Tetraethyl lead (TEL) has a common application as a gasoline additive, due to which it is a source of heat and electricity [244]. It exists in two states: Pb^2+^ and Pb^4+^ [245]. Its toxicity determines its bioavailability as well as mobility in the soil. Its common forms are oxides, hydroxides, ionic form, metal oxyanion complexes [200], phosphates, and carbonates (at pH above 6). The stable and insoluble forms include oxides, sulfides, and pyromorphites [246]. Its exposure occurs by food, water, and inhalation, which affects the circulatory, gastrointestinal, reproductive, neurological, muscular, kidney, and genetic systems [123]. Dose and exposure time are prime factors [247]. The permissible level of Pb in drinking water is <10 µL/L [230].

#### Bioregulation of Lead by *Bacillus* spp.

**Figure 5:**
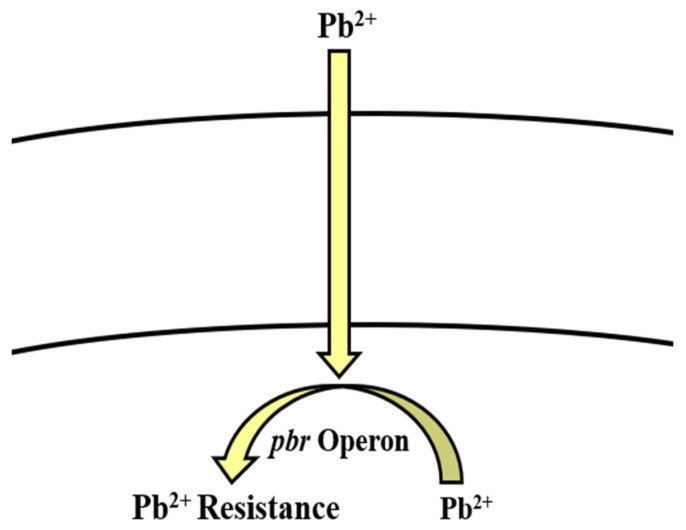
*pbr* operon involved in the regulation of lead resistance in *Bacillus* spp.

Pb-resistant *Bacillus* spp. have been reported previously [245]. *Bacillus* uses *pbr* operon [166,167,168] and active transport [248] as potential strategies to combat the toxic effects of Pb (Table 2, Figure 5). Microorganisms immobilize it by adsorption, chelation, inorganic precipitation, complexation, and biosorption. These processes involve bacterial cell wall functional groups including phosphate, carboxyl, carbonyl, sulfhydryl, and hydroxyl groups, which confer a negative charge to the cell wall. Binding of Pb to any of them results in insoluble substance. On the outside environment, Pb^2+^ is exchanged by Na or K cations [124]. Another method is adsorption through the cell wall, as it is comprised of organic macromolecules including polypeptides, polysaccharides, and proteins, which have the ability to adsorb Pb via electrostatic forces including Van der Waal’s forces, covalent or ionic bonds [124]. Pb interferes with microbial growth, morphology, and biochemical activities by damaging the DNA, protein, and lipids and even replacing the essential ions within the enzymes [123,249]. Microbes resist Pb toxicity by extracellular precipitation, exclusion, volatilization, biomethylation, cell surface binding, intracellular sequestration, and enhanced siderophore production [123]. Much like other Gram-positive bacteria, *Bacillus* spp. also employ one or several of these methods to remove Pb from the contaminated environments [125,126,127] (Table 1). 

### 5.6. Copper

Copper (Cu) is categorized into the group I-B, and period 4 of the periodic table [200]. It is a soft, diamagnetic, malleable, and ductile metal with remarkable electrical and thermal conductivity. It acts as a soft and intermediate Lewis acid and tends to bind to soft bases (hydride, alkyl, thiol, phosphine) and auxiliary ligands to Cu^2+^, such as sulfate and nitrate [250]. Apart from being widespread in the environment thanks to anthropogenic actions, it also exists naturally in the form of minerals such as sulfides, carbonates, and oxides. The discovery and use of Cu dates back to ancient times, with its use spanning more than five thousand years. It exists in either of its two oxidation states, which can be the oxidized, divalent cupric form (Cu^2+^) or the reduced, monovalent cuprous form (Cu^+^) [251]. The significance of Cu in biological systems is pivotal; it serves an important role as a micronutrient in several biological processes in both prokaryotic and eukaryotic organisms. However, this stands only for lower concentrations of the metal; higher concentrations tend to induce cell toxicity, resulting in intracellular damage including changes in DNA, respiration, and overall growth [252]. Moreover, Cu is essentially required as a co-factor in more than thirty known enzymes, due to its ability to reversibly interconvert from its less to more required forms very easily [253]. Elevated levels of Cu exposure are a deep-rooted cause of environmental pollution by Cu, the fundamental reason being anthropogenic processes. Industries using Cu or its compounds, Cu mining, burning of fossil fuels, inadequate treatment of wastewater, accumulation in dumps, production of phosphate-containing fertilizer, and natural processes such as erosion, volcanic eruptions, forest wildfires, and decay are all processes which greatly contribute to its presence in the environment. Furthermore, its production is also a source of direct Cu pollution, capable of harming the fragile ecosystems of soil, water, and air, respectively [254].

#### Bioregulation of Copper by *Bacillus* spp.

**Figure 6:**
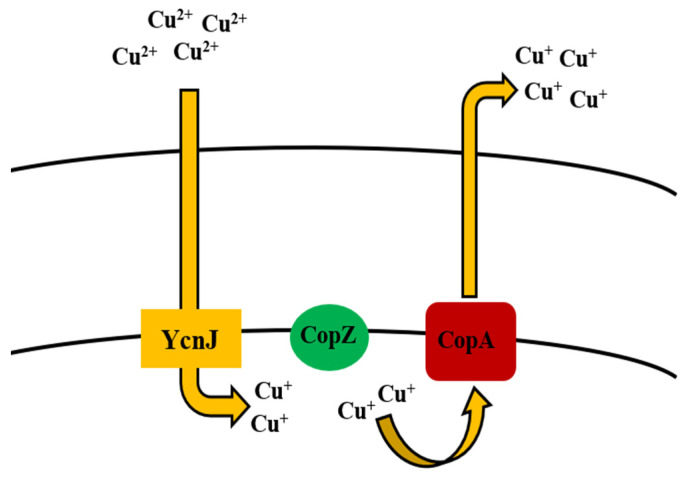
Uptake and efflux mechanism of copper by *Bacillus* spp.

Like many other heavy metal ions, Cu is considered to be essential for *Bacillus subtilis*, while concentrations exceeding normal amounts can be toxic for the cells. Species like *B. thuringiensis*, *B. cereus*, *B. licheniformis*, and *B. sphaericus* are also involved in the removal of Cu, when their concentrations exceed the required limit [117,128,129,130,131,132,133,255,256] (Table 1). In correlation with other bacterial species, Cu in the cytosol is regulated by CueR [257]. *B. subtilis* CueR is responsible for regulating the *copZA* operon which encodes both Cu chaperone and a P-type ATPase for Cu efflux, the latter of which is a member of the integral family which exports metal out of the cell [169]. In the former, CopZ plays a key role as Cu chaperone in transferring Cu over to CopA [170], which contributes to uptake of Cu, while CopB is accountable for Cu efflux and detoxification [171] (Table 2, Figure 1). Chillappagari et al. [172] reported that YcnJ was associated with Cu uptake function of *copZA* operon in *B. subtilis*, where their model proposed that the protein works in conjunction with other Cop proteins to facilitate Cu transport in and out of the cell (Figure 6).

### 5.7. Chromium

Chromium (Cr) is the 7th most abundant element on Earth, found widely in its crust. It belongs to the group VI-B in the periodic table and is characterized as a redox transition metal (3d) with variable valences (−2 to +6). According to the WHO and US EPA, the permissible limit of Cr in drinking water is >50 μg/L [258], whereas in soil, concentrations of >90 mg/kg are considered to be safe [259]. Among its many oxidation states, Cr mainly exists in its trivalent (Cr^3+^) and hexavalent (Cr^6+^) ions which are the most stable forms found naturally [260]. The toxicity and absorption are primarily dependent upon the oxidation state of the metal, which is also affiliated with its concentration in biological systems [261]. The hexavalent form is reported to be relatively more toxic than the former, which remains fairly innocuous and is associated with lipid and sugar metabolism [262]. On the other hand, Cr^6+^ has been reported to act as a carcinogen and a mutagen, which is absorbed readily into the human food chain [263]. Cr^3+^ has a greater affinity for organic solutions and is able to form hydroxide, oxide, and sulfate complexes in nature, while Cr^6+^ tends to persist more in the environment and biological systems owing to its emission due to anthropogenic actions [264]. Furthermore, Cr^6+^ occurs as a potent oxidizing agent, as part of many solutions, but it is most commonly found in the form of oxyanion chromate (Cr_2_O_4_^2−^), which is the only ion present in pH < 7. Compounds of Cr^6+^ pose a significant risk as environmental contaminants due to their elevated toxicity, as high concentrations lead to changes in the structure and variety of microbial systems present in various environments [265]. Nevertheless, Cr^3+^ appears to be accountable for most of the Cr toxicity at intracellular levels. Concentrations of Cr in the environment are imputed to anthropogenic actions, from their production by various industries, such as tanning, electroplating, chemical industry, smelting, and the leather industry [259], to being discharged into the environment in the form of untreated residues, wastewater, and effluent sludge. Moreover, the mining of Cr from its ores also contributes greatly to its environmental burden, threatening many fragile ecosystems [266].

#### Bioregulation of Chromium by *Bacillus* spp.

**Figure 7:**
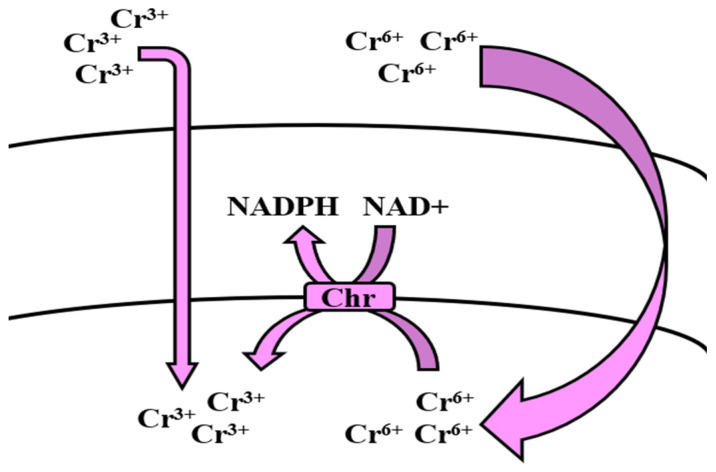
Uptake mechanism of *Bacillus* spp. for cadmium and its regulation.

The removal of Cr from contaminated environments has been achieved in the past through many physico-chemical techniques. However, one of the most efficient processes remains microbial bioremediation, as most microorganisms tend to survive even in highly heavy metal-contaminated environments. Moreover, bioremediation can be utilized to treat and remove these ions in order to combat environmental pollution, which rings true for most of the microbes used to treat Cr^6+^ which are isolated from tannery effluents and sewage [267]. Cr^3+^ is relatively non-toxic, compared with Cr^6+^, and is insoluble, and is therefore much easier to remove through precipitation, whereas Cr^6+^ tends to persist in nature [268]. Microbes in Cr-contaminated environments have inherent resistance which enables their survival by evasion of metal stress via metal efflux, uptake, or detoxification through reducing immobilization of metal ions [173] (Table 2). Among the methods of microbial biodegradation, enzyme-regulated biotransformation of Cr^6+^ from its toxic to non-toxic state (Cr^3+^) by bacteria is considered to be a cheap and efficient method of Cr removal from contaminated soil and wastewater. In Gram-positive and negative bacteria, this reduction of chromate is arbitrated by an enzyme, ChrR (chromate reductase), which is exclusively found in Cr-resistant bacteria and is not often affiliated with plasmids [174]. This reduction can be mediated both aerobically and anaerobically, through the cytosolic component and membrane-bound component, respectively [175]. However, in *Bacillus* spp., reduction of chromate ions is regulated through the aerobic process, which transpires through the transfer of electrons from the hexavalent to trivalent form of Cr; this occurs through the formation of an unstable intermediate (Cr^5+^), regulated by NADH/NADPH [269] (Figure 7). *B. licheniformis* was reported to remove 95% and 69.4% of Cr in various studies [138,141,270]. Nayak et al. [135] reported that *B. cereus* removed more than 81% of Cr. Other *Bacillus* spp. have also been reported to be effective in mitigating Cr pollution [121,136,137,139,140,142,143,144,271,272] (Table 1).

### 5.8. Mercury

Mercury (Hg) is a shiny, silvery metal at room temperature, which belongs to Group XII and is the 80th element in the periodic table [273]. It is one of the most toxic elements on Earth with adverse health effects to all living beings including humans. It is traditionally grouped together with lead and cadmium for the “big three heavy metal poisons” which are not reported to be involved in any essential biological function [200]. Once introduced into various environments, Hg tends to accumulate rapidly, with it being cumulatively present in soils, sediments, water, inside living things, and in the atmosphere. Worldwide, levels of Hg pollution have greatly increased at atmospheric level due to the various mining and industrial processes through which the metal can be released into the nearby soils and sediments [274]. Due to its tendency to accumulate in the ecosystem, it can remain suspended in the atmosphere for a time period of approximately 24 months [275]. Major anthropogenic activities contribute more significantly to Hg release than natural processes, which can aid in its transmission worldwide [274]. Further aiding its spread, Hg exists naturally in its elemental (Hg^0^), organic, and inorganic (Hg^1+^, Hg^2+^) forms, which are interconvertible in different environments, due to which it can insert itself deep into sources of soil, sediment, air, and water, respectively [276]. When elemental Hg is released into the environment, it tends to make small, tightly packed, sphere-shaped droplets in a process known as vaporization, due to the massive surface tension and vapor pressure [277]. On the other hand, the inorganic forms of Hg are more ubiquitously found, due to their economic significance and widespread applications in several industries which are the major sources of Hg emission into the environment [278].

#### Bioregulation of Mercury by *Bacillus* spp.

**Figure 8:**
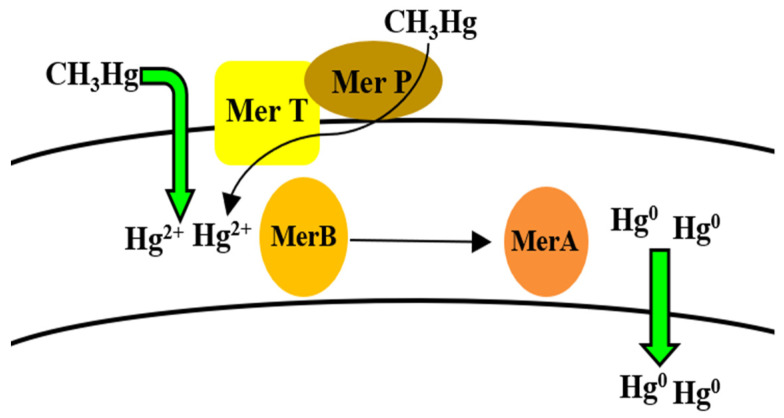
Uptake and reduction mechanisms for mercury by *Bacillus* spp.

*Bacillus* and its various species have been positively associated with the bioremediation of Hg, as reported by various studies conducted over the years [145,146,147,148,149,150,279] (Table 1). The high toxicity of Hg leads to its dangerous effect on biological systems. Faced with high concentrations of toxic Hg, bacteria are equipped with several mechanisms in order to ensure their survival. The presence of mercury resistance genes and operons, particularly the *mer* operon, are reported in both Gram-positive and negative bacteria [208]. In bacteria, two types of operons exist for resistance against Hg, with one being a narrow spectrum *mer* operon and the other being a broad-spectrum operon. The reversible detoxification of Hg from its toxic to non-toxic state is induced inside the cell, but the egress of Hg outside the cell is facilitated by diffusion [232]. Once the metal ions are out of the cell, they can again undergo oxidation by other bacterial species [160]. This phenomenon has been investigated in many bacteria, where its activation via MerR, an activator protein, is responsible for the detection of Hg. The reduction of inorganic Hg^2+^ and organic Hg is facilitated through the enzyme mercuric reductase and the lyase enzyme, encoded by the *merA* and *merB* gene, respectively [176] (Figure 8). Furthermore, the different forms of Hg may serve a function in its regulation of transportation, and bioaccumulation, which may be a cause of Hg toxicity in habitats and ecosystems near sites where Hg is mined or emitted [280]. Apart from Hg transformation by enzymes, it can be bioremediated by the action of metallothioneins by accumulating Hg ions in an inactive form [177] (Table 2). Though the mechanism of Hg resistance is far better understood in Gram-negative bacteria, Gram-positive bacteria also share the same *mer* operon, although some similar genetic sequences and some disparity among bacterial species is widely reported. In *B. cereus*, *merA* gene was identified in the USA which is usually reported among *Bacillus* group [178,179]. This occurrence among *Bacillus* spp. has been attributed to the location of these genes on transposons [281]. *Bacillus* spp. isolated from soils were also reported to possess Hg-resistant genes which facilitated the reduction and eventual bioremediation of Hg [180]. Furthermore, *B. cereus* was genetically engineered to harbor Hg resistance via a *mer* operon from another *Bacillus* sp., *B. thuringiensis*, for better biosorption and volatilization of Hg from contaminated sites [177].

### 5.9. Manganese

Manganese (Mn) is a transition metal belonging to Group VII (atomic number 25) of the periodic table, and is the 12th most abundantly found element on Earth [282]. It is widely distributed in nature as an essential trace element significant to all living beings. In the environment, Mn is not found as a free element, rather as a component of different naturally found minerals in the form of various oxides [283]. Mn is found in several oxidation states, of which the divalent form is reported to be the most common [284]. It is also able to exhibit all valences from 1 to 7, though 1 and 5 are very rare [185]. The essentiality of Mn lies in its significance as a co-factor and regulator of various processes in biological systems. Therefore, its toxicity levels can be low in living beings, but unusually high concentrations can cause detrimental effects to the nervous system in humans [186]. Many minerals are comprised of Mn nodules, which can be found in various environments such as soil, sediment, and in water, depending on geographical position, which is also a deciding factor for the size of the nodules. There are many industrial applications of Mn, making Mn the 4th most used metal in industries on the basis of tonnage. Mn is most used in the steel industry, followed by the chemical and battery industry, in which more than 90% of the world’s Mn is utilized in the desulfurization and reinforcement of steel [282].

#### Bioregulation of Manganese by *Bacillus* spp.

**Figure 9:**
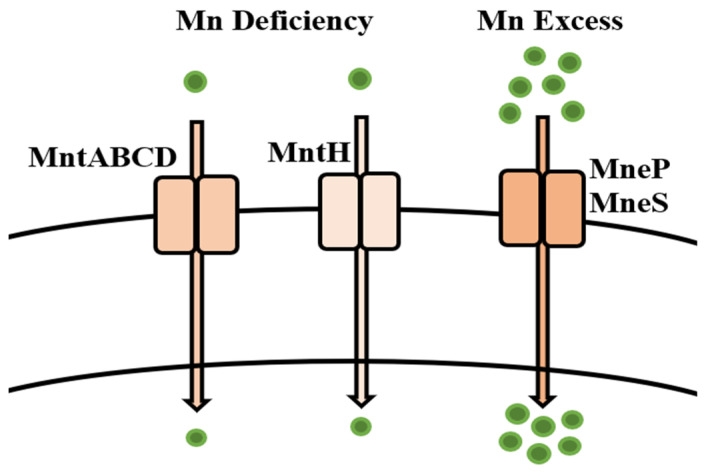
Uptake mechanisms of *Bacillus* spp. for the regulation of manganese inside bacterial cell.

The essentiality of Mn mitigates the overall toxicity of the metal in biological systems, although some toxicological conditions can be observed in adults and children alike, due to high Mn concentrations [187]. However, Mn found in high concentrations in the environment is difficult to degrade. Usually, the removal methods for Mn involve chemical techniques and the addition of chemical reagents which oxidize Mn by oxidation or aeration from the contaminated sites, but these methods are deemed to be very expensive, and not very efficient. The use of these methods to remove Mn from the environment also leads to the production of secondary metabolites [188]. The removal and regulation of Mn by microorganisms is mediated by metal-specific regulators that regulate low or sufficient concentrations of Mn inside the cell. This system is well characterized in bacteria, especially Gram-positive bacteria such as *Bacillus* spp., due to the fact that many studies have reported Mn bioremediation by *Bacillus* [44,151,152,153], whereas the Mn homeostasis mechanisms have been studied extensively over the years (Table 1). In *B. subtilis,* Mn concentration is regulated by MntR, an Mn-specific metallo-regulator related to the DtxR/IdeR family [181], which serves important roles in the sensing of metals such as iron (Fe) and Mn in several bacterial species [189]. It has also been suggested by Helmann in his review [285] that MntR is not only involved in the regulation of Mn uptake, but also in some conditions can sense and regulate Fe concentrations and their eventual homeostasis in the cells. *B. subtilis* is reported to require Mn for growth and to regulate free and labile concentrations of Mn inside the bacterial cell. Moreover, the uptake of Mn into Mn-starved *B. subtilis* cells demonstrated an ephemeral inhibition in growth, which resumed only after the concentration of the metal inside the cell reverted to normal [182]. In conditions of Mn limitation, *B. subtilis* MntR is de-repressed, which leads to the expression of two Mn uptake systems [183], namely the ATP-dependent Mn ABC transporter (*MntABCD* operon), and MntH, a proton-coupled symporter [184] (Table 2). When there is an excess of Mn in bacterial cells, MntR regulates the expression of Mn efflux pumps, MneP and MneS [286]. Under conditions of extreme excess of Mn in *B. subtilis* cells, the yybP–ykoY riboswitch is activated inside the cell, which senses the excessive concentrations of Mn and in turn activates Mn-sensing genes that regulate homeostasis in the cell [203] (Figure 9).

### 5.10. Molybdenum

Molybdenum (Mo) is a member of Group VI of the periodic table which also houses similar metals such as chromium and tungsten. Though Mo has been characterized as a metal since the Middle Ages, its pure form was produced for the first time in 1893 [287]. Mo is not naturally found in its metallic state, rather it is found in conjunction with other elements. It is naturally found in minerals, soils, rocks, and water. In high or moderate concentrations of Mo, the metal readily forms various complexes comprising of polymolybdates. In its natural state, Mo occurs as a silvery-white metal which in its powder form can give off a black or greyish hue [287]. Mo exists in oxidation states ranging from 2- to +6, though it is significant in states of +4, +5, +6. It is an essential trace element required for biological life and evolution. The different oxidation states of Mo are important as they partake in various redox reactions and act as cofactors for different enzymes. Nevertheless, exposure to Mo can cause toxicity, which can result in several health defects in biological systems [190]. The industrial applications of Mo are extensive, such as its usage as an additive substance in commercial lubricants, catalysts, corrosion inhibitors, and as a vital component in tungsten production. Most of the Mo produced is used in the steel and welding industry, where it is used for the reinforcement of steel and other alloys [288]. In analytical chemistry, Mo has long been used in several tests and techniques as a colorimetric agent. The natural and anthropogenic processes that contribute to the introduction of Mo into the environment include soil leaching, soil run-off, sedimentation, as well as burning of fossil fuels, mining, and mine wastes [289].

#### Bioregulation of Molybdenum by *Bacillus* spp.

In bacteria, the transportation of Mo may be facilitated by sulfate transport systems, but the major uptake system has been characterized as the inducible ABC-type transporter (*modABC* operon), which is also able to facilitate the uptake of tungsten [160,290] (Figure 1). This transport system has been reported to regulate the uptake (high affinity) of Mo from the environment [291,292], and has been employed by *Bacillus* spp. to uptake Mo [154,155] (Table 1). The main protein of this system is ModA, which is a substrate-binding periplasmic protein primarily interacting with other proteins of the system such as ModB and ModC that facilitate the transport of Mo via hydrolysis of ATP [293]. This system has been well characterized in Gram-negative bacteria such as *E. coli*, though similar *mod* genes have been identified in many bacterial species, including *B. subtilis* [294,295] (Table 2). Moreover, high molybdate affinity uptake has been reported to be mediated by ModA protein in *B. subtilis* in Mo-limited conditions [296].

### 5.11. Gold

Gold (Au) resides in group XI of the periodic table, with an atomic number of 79. It is one of the rarest elements on this Earth, and its inertness allows it to exist in mineralized zones where it may exist in one of its many well-characterized geological forms [297]. It is categorized as a non-essential element, incapable of forming free ions in aqueous solutions and highly toxic to living beings under high concentrations [191,298]. Naturally found Au is usually present in the form of alloys with other indigenous metals. Under surface conditions, Au is found in the form of metal colloid, aurous, and auric forms, with valences of 0, +1, and +3, respectively, whereas its state is dependent upon the thermodynamic reactions taking place in its presence. Moreover, Au readily forms complexes with ligands which are organic in nature, a trait synonymous with its native group in the periodic table [297].

#### Bioregulation of Gold by *Bacillus* spp.

Though the resistance mechanisms of Au uptake and efflux in Gram-negative bacteria such as *Cupriavidus metallidurans* are well known [191,299], the mechanisms are poorly understood in Gram-positive bacteria. Nevertheless, they are capable of mediating precipitation and biomineralization of Au complexes in natural environmental settings [300] (Table 2). This role offers some insight for microbes in mediating Au mobility and promoting bacterioform and secondary Au grains [301]. As is the case with other metals, complexes of Au also tend to be toxic for bacterial species at high concentrations, which may lead to the generation of free radicals and disruption of enzyme activity [302]. Microbe-mediated solubilization of Au is dependent upon the microbe’s oxidation ability, as well as its ability to facilitate ligands which can then bind to Au ions for their stability via complex or colloid formation. This phenomenon is true for many bacterial species, which reside in different environmental settings. In particular, heterotrophic bacteria have been linked to Au solubilization in soils rich in organic matter. *B. subtilis*, along with other *Bacillus* spp., has been previously reported to solubilize Au as Au-amino acid complexes [303,304]. Moreover, it was observed that the reduction conditions generated by ETC of bacteria resulted in the precipitation of Au on an extracellular level, along with the formation of iron sulfide by hydrogen sulfide, causing Au removal from solution through the process of reductive adsorption [305]. Very early studies on *B. subtilis* do report this extracellular reduction of Au from chloride solution, as an outcome of selective adsorption [306]. Some other studies also report the accumulation of Au by *Bacillus* spp., which is inherently possible due to the cell membrane structure and its bound proteins [192,307]. Apart from the cell membrane, the enzyme-mediated hydrolysis of ATP in *Bacillus* spp. also plays a significant role, as the regulation of intracellular metabolism has been reported to be associated with the bioaccumulation of Au [191,307].

### 5.12. Silver

Silver (Ag) is a silvery-white metal which is found naturally in its metal state as well as in ores. The metal itself is insoluble in water but becomes soluble once bound to salts such as nitrates. Some compounds of Ag are stable in air and water, but the rest appear to be sensitive to light. In its natural form, Ag is found with sulfide or closely bound in association with other metal sulfides [308]. With two stable isotopes, it exists as a monovalent ion but readily forms complexes with other metals. Seldom occurring as a singular metal, it is often recovered from the environment from its ores, whereas the secondary production is generated from old Ag scrap and Ag-containing products, such as photo-films, wastes, batteries, jewelry, coins, and dental equipment. Atmospheric emissions are attributable to smelting procedures, manufacturing and recycling of photographic films, combustion of fossil fuels, as well as cloud seeding [309]. Along with the environmental effects of Ag in the environment, the anti-bacterial and biological use of Ag and its nanoparticles have been extensively researched for many years. The most widely known use of Ag in medicine is its use as a topical antimicrobial agent for the treatment of burns, which has since translated into its usage in many clinical dressings, eye drops, and even dentures [310].

#### Bioregulation of Silver by *Bacillus* spp.

In the course of removing Ag or surviving amidst Ag in the environment, bacterial species display high sensitivity to Ag due to its probable non-specific toxic nature, and its ability to permeate and affect bacterial metabolism, transport, and ion exchange systems [311]. Therefore, bacterial resistance to Ag and its mechanisms usually is dependent on its binding proteins and affiliated efflux pumps in Gram-negative and/or positive bacteria [193,194,195]. Molecular bioregulation and resistance mechanisms active against Ag in Gram-negative bacteria are well studied, with two Ag efflux systems (SilCBA and SilP) active in transporting Ag out of the cell while the mechanisms in Gram-positive bacteria, to the best of our knowledge, are poorly elucidated, with reports of only MRSA isolates demonstrating resistance against Ag, courtesy of *Sil* genes [196,197,198] (Table 2). However, one study reported the maximum uptake (73.6 mg g^−1^) of Ag by *B. licheniformis* from aqueous solution, which denoted an active uptake system in *Bacillus*, mediated either through efflux pumps or resistance genes [156] (Table 1). 

## 6. Conclusions

Biological methods, particularly bacterial-mediated bioremediation, are a cost-effective, feasible, and environmentally friendly approach for the treatment of contaminated soil and groundwater, industrial wastewater, effluents, and sludge. Many bacterial species, such as *Bacillus* spp. are viable options for bioremediation, as their molecular mechanisms are suggestive of the resistance, transport, efflux and detoxification systems that work in high and low concentrations of heavy metals to maintain and regulate metal homeostasis at the cellular level. In order to utilize these mechanisms for the efficient removal of contaminants, including heavy metals, further research is required in order to study and characterize previously unknown molecular mechanisms of resistance, which can aid in elucidating the role of *Bacillus* spp. in bioremediation at a much deeper level.

## Figures and Tables

**Table 1:** Uptake ability of *Bacillus* spp. for various heavy metals.

Metal	Bacterial Strains	Initial Metal Concentration (%/mg/g/mM/ppm/mg/L)	Metal Uptake Ability (%/mg/g/mM/ppm/mg/L/mol/g)	Reference
Arsenic	*Bacillus* sp. KM02	100 ppm	51.45% (As^3+^)	[59]
	*B. licheniformis* *B. polimyxa*	0–100 mM 0–20 mM	100 ppm (As^0^) 100 ppm (As^0^)	[88]
	*Bacillus* sp. IIIJ3–1	350 smM (As^5+^) 10 mM (As^3+^)	350 mM (As^5+^) 10 mM (As^3+^)	[71]
	*B. barbaricus*	-	20 mM (As^5+^) 0.3 mM (As^3+^)	[91]
	*B. indicus* Sd/3T	0 mM 0 mM	20 mM (As^5+^) 30 mM (As^3+^)	[89]
	*B. selenatiredreducens*	10 mM	0 mM (As^5+^) 0.3 mM (As^3+^)	[87]
	*B. arsenicus* con a/3	20 mM 0.5 mM	20 mM (As^5+^) 0.3 mM (As^3+^)	[90]
	*B. cereus* W2	50 mg/L	1.870 mg/L (As^3+^)	[86]
	*B. cereus* EA5 *B. fusiformis* EA2	15 mg/L	94.9% 99.7%	[84]
	*B. arsenicus* MTCC 4380	2000 mg/L 1800 mg/L	89.462% (As^5+^) 83.043% (As^3+^)	[92]
Zinc	*B. subtilis*	178 mg/L	49.7 mg/L	[105]
	*Bacillus sp.* (KF710041) *B. subtilis* (KF710042)	-	73.29% 78.15%	[106]
	*B. licheniformis*	-	53%	[107]
	*B. cereus*	0–200 mg/L	66.6 mg/g	[108]
	*B. jeotgali*	75 mg/l	30%	[109]
	*B. subtilis* D_215_	100 mg/L	63.73%	[110]
	*B. firmus*	100 mg/L	61.8%	[111]
	*B. altitudinis*	100 mg/L	87 mg/L	[112]
Nickel	*B. subtilis*	2.14 ppm	85.61%	[113]
	*B. subtilis* BM1 *B. subtilis* BM2 *B. subtilis* BM3	2–32 mg/L	98.54% 99.2% 96.3%	[114]
	*B. subtilis*	178 mg/L	57.8 mg/g	[105]
	*Bacillus* sp. KL1	100 ppm	55.06%	[115]
	*B. thuringiensis* KUNi1	0–7.5 mM	82%	[116]
	*B. thuringiensis* OSM29	25–150 mg/L	94%	[117]
	*B. thuringiensis*	250 mg/L	15.7%	[118]
Cadmium	*B. safensis*	40 ppm 60 ppm	83.5% 98.10%	[119]
	*B. licheniformis*	-	98.34%	[120]
	*B. catenulatus* JB-022	150 mg/L	66%	[121]
	*B. thuringiensis* DM55	0.25 mM	79%	[122]
Lead	*B. pumilus* MF472596	100–1000 ppm	96%	[123]
	*B. subtilis* X3	200–1400 mg/L	590.49 mg/g	[124]
	*B. cereus*	5–100 mg/L	36.71 mg/g	[125]
	*Bacillus* S1 *Bacillus* SS19	75 and 100 mg/L 50 mg/mL	53%, 51% 57%	[126]
	*Bacillus* sp. AS2	500 ppm	74.5 mg/g (99.5 %)	[127]
Copper	*B. cereus*	100 ppm	54%	[128]
	*B. cereus*	400 ppm	48%	[129]
	*B. thuringiensis* OSM29	25 mg/L	91.8%	[117]
	*B. licheniformis*	5 gm/L	32%	[130]
	*B. thioparans*	40 mg/L	27.3 mg/g	[131]
	*B. subtilis* D_215_	100 mg/L	67.18%	[110]
	*B. sphaericus* *B. cereus* *Bacillus sp.*	17.6 mg/L 44.0 mg/L 88.0 mg/L	5.6 mol/g 5.9 mol/g 6.4 mol/g	[132]
	*Bacillus* sp. SG-1	-	60%	[133]
Chromium	*B. cereus* NWUAB01	100 mg/L	43%	[134]
	*B. cereus*	100 mg/L	81%	[135]
	*B. salmalaya* 139SI	50 ppm	20.35 mg/g	[136]
	*B. cereus* FA-3	1000 μg/ml	72%	[137]
	*B. licheniformis*	15 mg/L	95%	[138]
	*Bacillus* sp. B	500–4500 mg/L	47%	[139]
	*B. marisflavi*	200 mg/L	5.783%	[140]
	*B. licheniformis*	300 mg/g	69.4%	[141]
	*B. thuringiensis*	250 mg/L	83.3%	[142]
	*B. licheniformis* *B. laterosporus*	-	62 mg/g 72.6 mg/g	[143]
	*B. circulans* *B. megaterium*	0.96 mg/L	34.5% 32%	[144]
Mercury	*B. thuringiensis* CASKS3	200 mg/L 400 mg/L 600 mg/L	62.4% 54% 40%	[145]
	*B. licheniformis*	50 mg/L	70%	[146]
	*B. cereus *BW-03(pPW-05)	5–50 ppm	96.4%	[147]
	*B. licheniformis*	100 μg/mL	70%	[148]
	*B. cereus *	5 mg/L	104.1 mg/g	[149]
	*Bacillus* sp.	1–10 mg/L	7.9 mg/g	[150]
Manganese	*B. thuringiensis* HM7	400 mg/L	95.04%	[151]
	*B. cereus* HM-5	600 mg/L	67%	[152]
	*Bacillus* sp.	13.3 mg/g	55.56 mg/g	[153]
Molybdenum	*Bacillus *sp. Zeid 14	-	200 mg/L	[154]
	*Bacillus* sp. strain A.rzi	0.1 mM	Not reported	[155]
Silver	*B. licheniformis* R08	100 mg/L	73.6 mg/g	[156]

**Table 2:** The proteins, operons, and methods of removal employed by *Bacillus* spp. for the bioregulation of heavy metals.

Metal	Protein(s)/Gene(s)	Method(s)	Reference
Arsenic	*ars* operon (*arsR*, *arsD*, *arsA*, *arsB*, *arsC*)	Reduction (Detoxification) Efflux Cell membrane binding, Adsorption on cell surface Complexation by exopolysaccharides	[59,81,87,99,100,101,102]
Zinc	Zur ZosA ycdHI-yceA yciABC CadA CzcD	Physico-chemical adsorption Ion exchange Efflux Uptake	[108,157,158]
Nickel	CzcD CitM	Efflux	[159,160]
Cadmium	*cad* operon *yvgW* KinA	Efflux	[122,134,161,162,163,164,165]
Lead	*pbr* operon	Efflux	[166,167,168]
Copper	CueR *copZA* operon (CopA, CopZ, CopB) YcnJ	Efflux by chaperone Uptake	[169,170,171,172]
Chromium	ChrR	Efflux, Uptake Enzymatic reduction (Detoxification)	[173,174,175]
Mercury	*mer* operon (*merR*, *merA*, *merB*) MerR MerA MerB	Efflux Enzymatic reduction (Detoxification)	[176,177,178,179,180]
Manganese	*mntABCD* operon MntR MntH MneP MneS	Efflux Uptake	[181,182,183,184]
Molybdenum	*modABC* operon	Uptake	[148,185,186,187,188,189,190]
Gold	Not reported	Bioaccumulation	[191,192]
Silver	SilP *sil* genes	Efflux	[193,194,195,196,197,198]

## Data Availability

Not Applicable.
